# S10-4 The Global Observatory for Physical Activity-GoPA! Policy Inventory: preliminary findings and lessons learned

**DOI:** 10.1093/eurpub/ckac093.053

**Published:** 2022-08-29

**Authors:** Željko Pedišić

**Affiliations:** Institute for Health and Sport, Victoria University, Melbourne, Australia

**Keywords:** physical activity, policy, monitoring, national, global

## Abstract

**Background:**

Insufficient physical activity (PA) is considered to be one of the main risk factors for premature mortality. Its high prevalence and associated costs for the health care system present a strong incentive for national governments to develop PA policies. There is a lack of recent and mutually comparable data on national PA policies, particularly for low- to middle-income countries. In 2019, the Global Observatory for Physical Activity (GoPA!) has therefore started collecting data on national PA policies globally using the GoPA! Policy Inventory, version 3.0. Our aim is to present the preliminary findings of this data collection and lessons learned during the process.

**Methods:**

The GoPA! Policy Inventory, version 3.0 was distributed to 149 GoPA! Country Contacts, with an option to provide their responses in an online survey or in an interactive Word document. The GoPA! Policy Inventory, version 3.0 includes 20 questions about availability, content, implementation, comprehensiveness, and effectiveness of national PA policies.

**Results:**

Data were collected for 24 high-income, 13 upper-middle-income, nine lower-middle-income, and three low-income countries (overall n = 49) from all six WHO regions. A large majority of countries (76%) reported having a national PA policy or plan. Fifty-seven per cent of countries reported having PA recommendations. However, less than a half of the countries (45%) had PA recommendations for each of the following key target groups: children and young people, adults, and older adults. National health surveillance/monitoring system, which includes measures of PA, was available in 78% of countries. Most Country Contacts indicated that the ministries of health, sport, and education had active roles in PA promotion. Quantifiable national targets for PA were established in 43% of countries. The median score for the implementation of policies was 6, for comprehensiveness five, and effectiveness four and a half (out of a maximum score of 10).

**Conclusion:**

Further efforts are needed in the development and implementation of comprehensive national PA policies. More countries should establish quantifiable national targets for PA and track progress toward meeting them. Monitoring the effectiveness and implementation of PA policies remains challenging, as countries do not have established systems for their assessment.

